# Supplementary material for: Identification and functional analysis of non-coding regulatory small RNA FenSr3 in Bacillus amyloliquefaciens LPB-18
Source: PeerJ. 2023 May 15;11:e15236. doi: 10.7717/peerj.15236 (PMC10194069; doi:10.7717/peerj.15236)
Supplement: Supplemental Information 4 [file peerj-11-15236-s004.zip › KO/CK-vs-T1_map/map05130.html]

KEGG PATHWAY: Pathogenic Escherichia coli infection - Reference pathway


|  |  |
| --- | --- |
| **Pathogenic Escherichia coli infection - Reference pathway** |  |

[
Pathway menu
| Organism menu
| Pathway entry
| Show description
| User data mapping
]

Enteropathogenic E. coli (EPEC) and enterohemorrhagic E. coli (EHEC) are closely related pathogenic strains of Escherichia coli. The hallmark of EPEC/EHEC infections [DS:H00278 H00277] is induction of attaching and effacing (A/E) lesions that damage intestinal epithelial cells. The capacity to form A/E lesions is encoded mainly by the locus of enterocyte effacement (LEE) pathogenicity island. Tir, Map, EspF, EspG are known LEE-encoded effector proteins secreted via the type III secretion system, which is also LEE-encoded, into the host cell. EPEC and EHEC Tir's link the extracellular bacterium to the cell cytoskeleton. Map and EspF are involved in mitochondrion membrane permeabilization. EspG interacts with tubulins and stimulates microtubule destabilization. LEE-encoded adhesin or intimin (Eae) is exported via the general secretory pathway to the periplasm, where it is inserted into the outer membrane. In addition to Tir, two potential host cell-carried intimin receptors, beta1 integrin (ITGB1) and nucleolin (NCL), have so far been identified. The distinguishing feature of EHEC is the elaboration of Shiga-like toxin (Stx). Stx cleaves ribosomal RNA, thereby disrupting protein synthesis and killing the intoxicated epithelial or endothelial cells.

|  |  |  |
| --- | --- | --- |
| Reference pathway | 184% 150% 122% 100% 82% 67% 55% | 图片下载 |
